# Maternal Preeclampsia and a Preterm Neonate With Small Bowel Stenosis, Volvulus, and Severe Intrauterine Growth Restriction

**DOI:** 10.7759/cureus.60901

**Published:** 2024-05-23

**Authors:** Evgeniya Babacheva, Ilias Chatziioannidis, Chrysostomos Kepertis, Papacharalambous Efthimia, Maria Lithoxopoulou, Kleanthis Anastasiadis, Maria Florou, Eleni Vasileiou, Christos Tsakalidis

**Affiliations:** 1 2nd Department of Neonatology and NICU, Papageorgiou General Hospital of Thessaloniki, Thessaloniki, GRC; 2 1st Department of Neonatology and NICU, Aristotle University of Thessaloniki, Ippokrateion General Hospital of Thessaloniki, Thessaloniki, GRC; 3 Department of Pediatric Surgery, General Hospital Papageorgiou, Thessaloniki, GRC; 4 Department of Pediatric Surgery, Aristotle University of Thessaloniki, Thessaloniki, GRC; 5 2nd Department of Pediatric Surgery, Aristotle University of Thessaloniki, Papageorgiou General Hospital of Thessaloniki, Thessaloniki, GRC; 6 2nd Department of Neonatology, School of Medicine, Aristotle University of Thessaloniki, Papageorgiou General Hospital of Thessaloniki, Thessaloniki, GRC

**Keywords:** sga, volvulus, bowel stenosis, intrauterine growth restriction, preeclampsia

## Abstract

Preeclampsia is a human-specific hypertensive disorder of gestation. It is associated with short-term adverse effects in the fetus and long-term complications in the neonate, mainly due to disrupted blood flow during critical periods of intrauterine development. An ischemic event in the uterus can affect many systems of the fetus, including a small bowel involvement. We present a case of a preterm, small for gestational age neonate with severe intrauterine growth restriction, small bowel stenosis, and volvulus without malrotation, born to a mother with severe preeclampsia.

## Introduction

Preeclampsia (PE) presents after 20 weeks of gestational age (GA) and is considered a complication of pregnancy. It is presented as an isolated new-onset arterial hypertension with blood pressure values ≥140/90 mmHg, occasionally superposed on chronic hypertension of the mother [1]. This disorder is divided into the following two common types: the placental PE presenting before 34 weeks of gestational age (GA) and the maternal PE occurring after 34 weeks of GA [2]. Although the etiology of PE remains unclear and multifactorial pathogenesis has been proposed, it is considered responsible for many short- and long-term negative effects for the mother and her offspring, with a high risk of morbidity and mortality [3]. The placental dysfunction, due to pathological remodeling of spiral arteries and ischemia, plays a key role in PE pathogenesis, as the maternal uterine blood flow is essential for supporting normal fetal growth and ensuring a normal intrauterine evolutionary process [4]. The intrauterine vascular insult resulting from placental dysfunction can affect many systems, including the gastrointestinal, and is proposed to be one of the most common pathogenic explanations for neonatal small bowel stenosis [5]. We herein report a preterm neonate with severe intrauterine growth restriction born of a mother with severe preeclampsia, who underwent surgical intervention for acute abdomen in the first days of life, with the postoperative diagnosis of small bowel stenosis and volvulus, without underlying intestinal malrotation.

## Case presentation

A preterm female infant was born at 28 weeks of gestational age to a primigravida, primipara 28-year-old mother and was directly admitted after birth to our neonatal intensive care unit (NICU) due to prematurity, respiratory distress syndrome (RDS), and severe intrauterine growth restriction. The family and maternal medical history before pregnancy were clear, reporting no chronic diseases, congenital anomalies, or hereditary conditions. The prenatal screening applied in the first and second trimester of pregnancy revealed normal findings.

The mother was admitted to the obstetric clinic of our hospital three weeks before delivery to receive treatment for arterial hypertension and undergo ultrasound-Doppler follow-up examinations. A baby girl was born via cesarean section following a severe episode of the mother’s preeclampsia, which caused anhydramnios attributed to placental insufficiency and abnormal blood flow, with rapid deterioration of velocity at the umbilical vessels, uterine artery pulsatility index (UtA-PI >95th centile), and middle cerebral artery (MCA <5th centile).

After delivery, the umbilical cord clamping was performed in the first minute of life while Apgar score was 5 and 7 at the 1st and the 5th minute after birth, respectively. The newborn was symmetrically small for gestational age (SGA), with a birth weight equal to 570 g (<3rd SD), a body length of 30 cm (<3rd SD), and a head circumference measured at 23 cm (<3rd SD). After the direct transfer to the NICU, her respiratory function deteriorated rapidly and the newborn was supported with synchronized nasal-intermittent positive-pressure ventilation and oxygen supplementation (max FiO_2_=0.5). The patient received two doses of surfactant in the first and sixth hours of her life because of RDS, and a rapid improvement in her respiratory status, along with a reduction in O₂ requirements, was observed.

**Figure 1:**
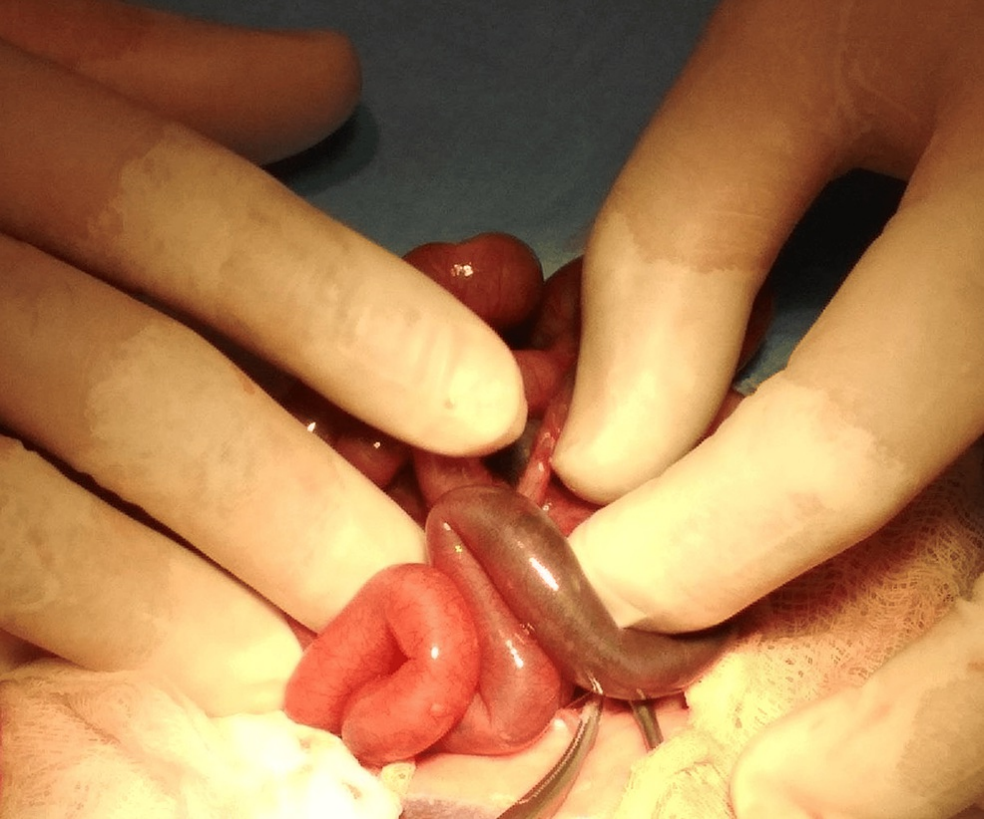
Intraoperative finding of ischemic lesion on the terminal ileum.

Total parenteral nutrition started soon after birth and on the second day of life (DOL) minimal enteral feeding was also applied with good tolerance. The meconium passing was noted on the second DOL. Neither metabolic nor electrolytic disorders were observed in the serum blood tests. However, on the fifth DOL, the newborn presented abdominal distention, bilious residuals in the nasogastric tube, and tenderness, as well as discoloration on the abdominal wall. Apart from these clinical findings, the laboratory and the radiological examinations were normal. On the sixth DOL, due to the gradual deterioration of its clinical status, a pediatric surgical consultation was requested and an exploratory laparotomy was decided. Intraoperatively, intestinal stenosis was found, with a length of 2 cm, located in the terminal ileum in direct contact with the ileocecal valve. Proximally to the stenosis, a prestenotic dilatation occurred with an adhering volvulus of a 15-cm-long ischemic loop, but without any bowel malrotation (Figure 1).

Resection of the terminal ileum and the ileocecal valve followed by an end-to-end ileo-colic anastomosis were performed. The postoperative period was uneventful and the enteral feeding with human milk was started on the 14th postoperative day. The enteral feeding was gradually increased and was well tolerated. The neonate was discharged from NICU, at three months of chronological age, in a very stable clinical condition, with a body weight of 2,195 kg (<3rd SD), a length of 43 cm (<3rd SD), and a head circumference of 33 cm (3-10rd SD). Later on, at the age of 12 months the infant was admitted to our hospital for poor weight-gain investigation, clinical and laboratory examinations were applied, without any concluding remarks. On the follow-up, at 18 months of life, the girl could walk independently. The Hammersmith Neonatal Neurological Examination score was still suboptimal (60/78) but improved impressively due to the early application of physiotherapy intervention services. Last but not least, the neurodevelopmental assessment at 24 months with the Bayley Scales III for cognitive, language, and motor domains was normal.

## Discussion

Hypertension during pregnancy is one of the main causes of maternal mortality and morbidity, associated with adverse effects on fetal wellbeing and intrauterine growth, along with short-term and long-term complications in the child’s life. Ηypertension during pregnancy can occur either before or after 20 weeks of gestational age, while preeclampsia (PE) presents after 20 weeks of GA [1]. PE is defined as maternal blood pressure ≥140/90 mmHg and is accompanied by one or more of the following conditions: proteinuria, maternal organ dysfunction, or uteroplacental dysfunction. PE occurring before 34 weeks of gestational age is defined as early (type I) and is of placental origin, while the onset after 34 weeks is considered late and is of maternal origin(type II) [2]. Early onset PE as a result of placental insufficiency is associated with severe vascular events consequently impacting the rate of intrauterine growth. A defective placenta probably results in the abnormal remodeling of the spiral arteries, leading to ischemia and the following increase of endothelial dysfunction factors, such as the soluble fms-like tyrosine kinase-1 (sFlt-1) [2,4].

The ultrasound, with the pulsed-wave Doppler umbilical images and the study of blood flow waveform, can detect and evaluate the placental vasculopathy effect on the fetus. Absent end-diastolic velocity (AEDV) or reversed end-diastolic velocity (REDV) in diastolic blood flow on waveforms can be detected in PE disease [6]. Neonates with reversed diastolic waveforms have restricted intrauterine growth and are placed at high risk for short-term and long-term negative effects. Intestinal complications, resulting from chronic intrauterine hypoxia, and blood redistribution to vital organs are a part of the pathological process associated with placental vasculopathy [6,7]. Intestinal atresia and stenosis account for about one-third of all neonatal cases of intestinal complications, mostly located in the small bowel [5]. The bowel resorption following an intrauterine, ischemic, intestinal event has been considered as the most probable etiological mechanism. The midgut volvulus is the most serious complication and a definite surgical emergency, due to bowel torsion, obstruction, and ischemia [8]. According to the literature, volvulus is usually associated with small bowel malrotation and abnormal mesenteric fixation, resulting in rotations and abnormal torsions during the period of intestinal development. Interestingly, in the present study, no malrotation was observed and the occurring volvulus was attributed to ischemic intestinal stenosis and extended prestenotic dilatation [9]. Fortunately, the other organ systems of the infant were not affected, especially the neurodevelopmental outcome was normal in the follow-up for our newborn. To conclude, intrauterine growth restriction is still a pathological condition that impairs the process of human growth and development early and later in neonatal life. Thus, the baby girl will remain in close monitoring of the potential consequences on the clinical, psychological, and neurodevelopment levels until adulthood [10].

## Conclusions

Pathological maternal conditions during pregnancy have a negative impact on fetal intrauterine growth, the immediate neonatal period, and long-term growth. PE remains until today, a clinical entity with unclear pathogenesis and a few not well-defined consequences. More prospective studies of pregnant females with PE and close monitoring are essential to increase the knowledge of the etiology and pathogenesis of such a condition like PE, in order to understand and prevent properly the potential severe complications on the fetus/newborn.
